# A pre-post study design: evaluating the effectiveness of a new community-based integrated service model on patient outcomes

**DOI:** 10.1186/s13033-024-00636-8

**Published:** 2024-05-09

**Authors:** Fabiana Engelsbel, René Keet, Annet Nugter

**Affiliations:** 1 GGZ Noord-Holland-Noord Research Department, Heerhugowaard, The Netherlands; 2 GGZ Noord-Holland-Noord FIT-Academy, Heerhugowaard, The Netherlands

**Keywords:** Recovery, Patient outcomes, Integrated mental healthcare, FACT, Community-based service model

## Abstract

This study aimed to evaluate a new service model, Specialists Together In the Community (STIC), in terms of patient outcomes. This model integrates Flexible Assertive Community Treatment (FACT)-principles with expertise of specialized teams that offer diagnosis-related outpatient treatment. In a pre-post design, symptoms and quality of life of 930 former FACT-patients were measured repeatedly pre- and post-STIC. Regarding patients in former specialized teams, pre- and post-treatment social functioning and symptoms were measured for the pre- (n = 944) and post-STIC (n = 544) groups. Against expectation, symptoms of former FACT-patients remained stable post-STIC compared to a slight decrease pre-STIC. According to expectation, pre- and post-STIC groups had an equal symptom reduction. Unexpectedly, the post-STIC group did not improve more on social functioning than the pre-STIC group. Explorative analysis showed less treatment contacts in the post-STIC group. The highly similar patient outcomes post-STIC could be improved by monitoring process outcomes and prolonging study duration.

## Introduction

In 2017, a new service delivery model in the Northwest area of the Netherlands was launched for people with severe mental illness, referred to as: “Specialists Together In the Community” (STIC). This model integrates principles of Flexible Assertive Community Treatment (FACT) teams with the expertise of specialized teams that offer outpatient treatment for diagnosis-specific groups. FACT is a well-defined model for integrated community mental health care, developed by Bähler and van Veldhuizen [24–26]. It was first implemented in the Northwest part of the Netherlands, in 2003. Since then, the number of FACT teams has grown rapidly throughout the country to nearly 300 teams in 2023, of which 233 are certified (https://ccaf.nl: 9-1-2023). In this study, the effectiveness of STIC, which may be considered a next step in the development of integrated community mental health care, will be addressed by investigating patient outcomes before and after its implementation.

FACT shares key characteristics with the Assertive Community Treatment (ACT) model, developed in the United States [20, 27, 29]. Both models use a multidisciplinary team-based approach, with teams including at least one psychiatrist, a psychologist, (specialist) psychiatric nurses, and social workers. These teams address a broad range of patient needs, involving illness management, rehabilitation, housing, and finances. The emphasis on the wide variety of patient needs as well as on social problems and functioning make both models compatible with a recovery-oriented approach [9], although the focus on recovery is more explicitly stated in the description of FACT [25]. Additionally, they both operate on the principle of shared caseloads, where team members collectively shoulder responsibility and provide assertive outreach services (i.e. visiting patient at home or another community setting) to patients at risk of relapse and/or hospital (re)admission. However, FACT serves a broader population of patients with severe mental illness, whereas ACT focuses only on the most severe patients (i.e. patients at risk of relapse and/or hospital admission). FACT employs two levels of care: individual case management for patients who are stable and require less intensive (multidisciplinary) treatment/support and a shared caseload approach with frequent outreach contacts when treatment needs to be intensified [24, 28]. This flexibility ensures the continuity of care within one single team.

Policy reforms in the Dutch mental healthcare in 2014 have fostered the further development and extension of FACT into STIC. These reforms divided the Dutch mental healthcare into primary (PMH: in Dutch Basis GGZ) and specialized mental healthcare (SMH: in Dutch SGGZ). Since then, patients with mild to moderate mental health problems are directed to either community social care teams, general practice mental health workers, and other PMH providers rather than specialized services. Previously, some of these patients would have received treatment in SMH, which now primarily focuses on highly complex, enduring conditions often characterized by comorbidity. Given this context, maintaining the organizational distinction within SMH between curative care (i.e. specialized cure programs) and FACT appears less relevant, since patients in specialized cure programs also present complex, long-lasting conditions ([19, 21]). To eliminate the aforementioned organizational distinction, we introduced STIC: a model in which the expertise from the specialized cure programs is incorporated into the FACT model. With the implementation of this model, more patients in the Northwest region of the Netherlands benefit from a wide range of recovery-oriented services tailored to their needs, including rehabilitation (e.g. Individual Placement and Support, IPS), integrated community support systems and disorder-specific interventions. Even more pronounced than in FACT, STIC is grounded in a vision of recovery: the notion that a majority of people can lead a subjective meaningful and satisfying life in their own community, despite their limitations caused by illness [1]. Recovery does not solely refer to the remission of symptoms (symptomatic recovery [2],), but also to regaining everyday functioning in work, social relationships and housing (social recovery), and to the view that well-being is achievable through a process of finding meaning, purpose and hope in one’s life (personal recovery).

Whereas the effectiveness of STIC has yet to be tested, overall, the implementation of FACT in the Netherlands has been associated with moderately positive patient outcomes and reduced hospital admissions [5, 6, 10, 15]. The effect of FACT on employment rates over time, however, has been more disappointing, with 10% of the patients remaining employed, 5% losing their employment, 3% gaining employment and 82% remaining unemployed [11]. However, the scientific evidence should be considered tentative due to the lack of good quality designs [13, 14], mainly due to practical considerations. For example, from 2008 it proved unfeasible to conduct a Randomized Controlled Trial (RCT) or even a less ‘strict’ comparison study, because mental health institutions were no longer willing to provide control groups receiving ‘care as usual’. Moreover, in the Netherlands there was no alternative model that could offer evidence-based interventions and recovery-oriented care in line with the recommendations of the Dutch Multidisciplinary Guideline for Schizophrenia [23, 27]. The broad use of (aforementioned) observational designs [5, 10, 15] is preferable, since it enables researchers to analyze data that yield more internally valid results than any other research design, even though positive effects could be overestimated as a result of patients improving naturally over time, regardless of the community model (i.e. regression to the mean).

In this study, an observational pre-post design was used to investigate the change in symptom- and social recovery, and quality of life as a result of the implementation of STIC. Distinct hypotheses were formulated for two groups of adult patients eligible for receiving SMH. Most patients in the first group receive long-term care. This group suffers from severe, complex and persistent mental health conditions, often associated with severe impairments in social and occupational activities. Prior to the implementation of STIC, this group of patients was treated in FACT teams. Approximately two-thirds of this group is diagnosed with a psychotic disorder, 15% with an affective disorder, 10% with a personality disorder, and the remaining 10% with other psychiatric disorders. It was hypothesized that as a result of the introduction of STIC, this patient group would benefit in terms of symptom recovery and quality of life due to the increased access to specialized treatment for certain, often comorbid, disorders, like trauma or depression. The second group of patients distinguishes itself both by main diagnosis and duration of care. The vast majority of these patients have been diagnosed with so-called common mental health disorders like depression, anxiety, post-traumatic stress disorder (PTSD), and personality disorder. Although a large percentage of these patients also reports serious problems in other areas of functioning such as work and interpersonal relations, they are not dependent on long term mental health care. Prior to the implementation of STIC, the focus of treatment was usually on symptom reduction of the main diagnosis as well as making the duration of treatment relatively short and finite. It was hypothesized that after the introduction of STIC, members of this group would have comparable symptom severity pre-and post STIC, and would benefit specifically in terms of social recovery due to improved access to social interventions, a core practice of FACT teams. With the focus remaining steady on specialized treatment, no change in symptom severity was anticipated.

## Hypotheses

### Long-term care group

1. Patients will improve more in terms of symptomatic recovery after implementation of STIC as compared to before.
2. Patients will improve more in terms of quality of life after implementation of STIC as compared to before.

### Short-term cure group

1. Patients with a completed treatment after the implementation of STIC will improve more in terms of social recovery as compared to patients with a completed treatment prior to the implementation of STIC.
2. Patients with a completed treatment after the implementation of STIC will improve equally in terms of symptomatic recovery as compared to patients with a completed treatment prior to the implementation of STIC.

## Methods

### Design

Data collection took place between September 2014 and September 2020. To test whether the long-term care group improved more in terms of symptom recovery and quality of life post-STIC than pre-STIC, a retrospective cohort study with repeated measurements before and after the implementation of STIC, starting on 01-09-2017, was used. A comparison was made between the pre-STIC course of symptoms and quality of life from September 2014 till September 2017 and the post-STIC course of these measures from September 2017 till September 2020.

To test whether the short-term cure group improved more after the implementation of STIC in terms of social recovery than pre-STIC, but comparable in terms of symptom recovery, an observational comparative pre-post design was used. In this design patients who had completed treatment prior to the implementation of STIC were compared with patients who had completed treatment after the implementation of STIC. Since most treatments of patients in the short-term cure group were of shorter duration compared with treatments in the long-term care group, it was not feasible to use the same design as in the long-term care group.

## Participants

### Long-term care group

Patients of former FACT teams located in the Northwest area of the Netherlands were included in the study if they: (i) were aged between 18 and 65 at the start of the study, (ii) had been in outpatient care during the whole study period for at least six consecutive years from September 1st 2014, and if they (iii) completed at least one measurement on one of the relevant questionnaires (see measurements). Patients were excluded from the study if they resided in a sheltered housing institution or were hospitalized. These groups differ from the majority of outpatients in terms of intensity of care and should therefore be analyzed separately. Due to small sample size such analyses could lead to biased results.

### Short-term cure groups

Patients of the former specialized outpatient teams were included in the study if they: (i) were aged between 18–65 at the start of their treatment, (ii) had completed their treatment within three years prior to the implementation of STIC (from here onwards: the pre-STIC group), or had completed their treatment within 3 years after implementation of STIC (the post-STIC group), (iii) completed the relevant questionnaire both at the start (pre-) and at the end (post-) of their treatment, and if (iv) the duration between their pre- and post-measurements was at least two weeks, within which they had at least two contacts with an health care professional.^1^

## Measurements

### Health of the nation outcome scales (HoNOS)

Psychosocial functioning was measured with the Dutch version of the HoNOS [12]. The HoNOS is administered by care practitioners and consists of 12 items, divided into four subscales: behavioral problems, physical and cognitive impairment, social functioning and psychiatric symptoms. The items can be scored from 0 “no problem present” to 4 “severe to very severe problem”. The total score is calculated by summing all item scores (ranged 0–48), with lower scores representing better psychosocial functioning. The Dutch version includes the following three additional items, namely “Manic symptoms”, “Treatment motivation” and “Compliance with medication”, resulting in a 15-item version. In this study a separate symptom scale was constructed in order to measure symptom recovery in the long-term care group. This scale included the following 14 items: “Substance abuse”, “Hallucinations and delusions”, “Depressed mood”, all subitems of item 8 “Other mental/behavior problems” (for more information about splitting item 8, see [7]), and “Manic symptoms”. The total score of this scale range from 0 to 56. The internal consistency of the 12-item version is reasonable (*α* = 0.78; [12]).

### Manchester short assessment of quality of life (MANSA)

Quality of life was measured with the Dutch version of the MANSA [18, 22]. This self-report measure consists of 16 items, of which 12 assess satisfaction with life as a whole, employment, financial situation, friendships, sex life, leisure activities, accommodation, personal safety, people that the person lives with, family relationships, physical health, and mental health. All items are measured on a seven point Likert scale, ranging from 1 “could not be worse” to 7 “could not be better”. The remaining four dichotomous (yes/no) items measure objective quality of life aspects, namely the existence of close friends, number of contacts with friends per week, accusation of a crime and victimization of physical violence. In this study, the total score of the 12-item version is used, ranging from 12 to 84, with higher scores indicating a better quality of life. The Dutch version of the MANSA has shown reasonable to good internal consistency for patients with a severe mental illness (*α* = 0.78–*α* = 0.82) [22].

### Outcome questionnaire-45 (OQ-45)

Symptoms and general functioning were measured with the Dutch version of the OQ-45 [4]. This self-report measure consists of 45 items that are scored on a five-point rating scale, divided into three subscales: symptomatic distress (SD 25 items), interpersonal relationships (IR 11 items), and social role (SR 9 items). Symptom recovery in the short-term cure groups is measured by the subscale SD, with scores ranging from 0 to 100. Social recovery is measured by the subscales IR and SR and have total scores ranging from 0 to 44 and 0 to 36, respectively. On all subscales, higher scores represent worse functioning. For the Dutch version the internal consistency is considered good for the SD and IR subscales (*α* = 0.91 and *α* = 0.80 respectively), and reasonable for the SR subscale (*α* = 0.78) [4].

## Statistical analyses

### Long-term care group

To examine the extent to which the findings were representative for all patients in the long-term group, patients with at least one completed HoNOS/MANSA were compared to those without any (completed) measurements in terms of socio-demographic and clinical characteristics. Independent t-tests were used for the continuous variables age and registration duration (in days), a Mann-Whitney test for the count variable number of comorbidities, and chi-square tests for the categorical variables gender, type of classification and comorbidity (yes/no).

To test the hypotheses, multiple two-level multilevel analyses were performed with Time as a first level and Patient as a second level variable. The Time variable was created by calculating the number of days between the start of the study on 01-09-2014 and the measurement date(s) (HoNOS/MANSA) within each person. In order to compare the pre- and post-STIC period, a ‘0’ was assigned to all measurements in the pre-STIC period and a ‘1’ for measurements in the post-STIC period. Further, the variable Time within period was created by calculating i) the number of days between the start of the study and the measurement date(s) in the pre-STIC period and ii) the number of days between the start of STIC and the measurement date(s) in de post-STIC period. To test if there was a change in the level of symptom recovery and quality of life post-STIC in comparison to pre-STIC, the interaction between Time within period and Period as well as their main effects were included in both analyses. A significant interaction would indicate that the course of the outcome variable (symptom recovery or quality of life) is different in the pre-STIC period than in the post-STIC-period, while a significant main effect of Time within period would mean that the outcome variable improved (if beta coefficient for the HoNOS is < 0, or > 0 for the MANSA) or deteriorated (if beta coefficient for the HoNOS is > 0 and < 0 for the MANSA) in the pre-STIC period.

An adjustment was made for one or more of the following variables, if their inclusion led to a better fit of the model: registration duration, gender, age and comorbidity (yes/no). These variables were included as main effects or as an interaction effect with Time, depending on whether their inclusion led to a better fit. Mean registration duration and mean age were centered around the grand mean.

### Short-term cure groups

To examine differences between the pre- and post-STIC groups, various clinical and demographic characteristics were compared. Independent t-tests were used for the continuous variables age and treatment duration, calculated as number of days between the first and last treatment contact. Mann-Whitney tests were performed for the non-normal distributed count variables representing different types of contacts (treatment, diagnosis, crisis, indirect,^2^no-show). If overdispersion was detected in these count variables (i.e. the observed variability is greater than what would be expected based on the assumed probability distribution), Generalized Linear Models (GLMs) with a negative binomial log-link were used instead, with overdispersion evaluated using the method outlined in Payne et al. [16]. Categorical variables including gender, type of classification and comorbidity were analyzed using multiple chi-squared tests.

We assessed the representativeness of the pre- and post-STIC groups separately by comparing the characteristics of those who completed both pre-and post-measurements with those who did not. Independent t-tests were used for the continuous variables age and registration duration. Chi-squared tests were performed on the categorical variables gender, type of classification and comorbidity. For the non-normally distributed variables concerning different types of contacts, the same statistical procedure was applied as described in the previous paragraph.

The hypotheses were tested by conducting several mixed ANOVA’s, with Time (pre-post measurement) as within-subject variable and Group (pre- and post-STIC group) as between-subject variable. The hypothesis regarding social recovery was tested by looking at the interaction between Time and Group on the SR and IR subscales separately. A significant interaction between Time and Group would indicate that the trajectory of the pre-STIC group differed from the trajectory of the post-STIC group. Specifically, a greater decrease from pre- to post-measurement scores in the post-STIC group compared to the pre-STIC group would support the hypothesis. In contrast, a non-significant interaction between Time and Group on SD would support the hypothesis regarding symptomatic recovery, which is expected to be comparable between the pre- and post-STIC groups. Bonferroni correction was applied (*α* = 0.017) due to multiple comparisons.

All abovementioned analyses were conducted in the statistical software program SPSS (Statistical Package for the Social Sciences) version 26.

## Results

### Representativeness of the long-term care group

Of the 949 included patients in the long-term care group, 930 (89%) had at least one measurement on the HoNOS/MANSA (= completers) and could therefore be used in the analyses (here forward: completers). Sample characteristics of the completers and the group without at least one measurement (here forward: non-completers; N = 19) are displayed in Table 1. Completers were predominantly male and, in most cases, diagnosed with a psychotic disorder. Mean registration duration was ± 13 years with an average spread of 6 years. More than forty percent of the patients had at least one comorbidity, with a mean number of DSM-V (Diagnostic and Statistical Manual of Mental Disorders 5th ed.) classifications of 1.6 (see Table 1).

**Table 1:** Demographic and Clinical Characteristics of Completers and Non-completers in the Long Term Care Group: Statistical Comparison with p-values

	Completers N = 930		Non-completers N = 19		Test results^2^
	% (n)	*M (SD)*	% (n)	*M (SD)*	
Gender (% male)	62.5 (581)		68.4 (13)		*p* = .811
Classification^**1**^					
Psychotic disorder Bipolar disorder Depressive disorder Personality disorder Other	67.2 (625) 11.4 (106) 7.6 (71) 6.6 (61) 7.2 (67)		63.2 (12) 10.5 (2) 10.5 (2) 0.0 (0) 15.8 (3)		
Comorbidity	43.3 (402)		31.6 (6)		*p* = .584
Registration duration in days		5617 (1994)		5167 (1892)	*p* = .355
Age		44.5 (10.4)		42.9 (10.1)	*p* = .621
Number of classifications		1.57 (0.77)		1.37 (0.60)	p = .274

^1^The difference between the groups in terms of DSM-V classification was not subjected to statistical testing due to the lack of observations in one of the non-completers' cells

^2^Test statistics are only reported for differences that achieve statistical significance

In comparison with the non-completers, completers were similar in terms of age, registration duration and number of DSM-V classifications. Also, there were no statistically significant differences in terms of gender and comorbidity. It was not possible to statistically test the difference between the groups in type of DSM-V classifications due to the absence of observations in one of the non-completers’ cells (see Table 1).

### Results of the long-term care group

Table 2 presents results of the final ML model with Symptoms (HoNOS) and Quality of Life (MANSA) as outcome variables. A statistically significant negative association between Time in Period and Symptoms suggested a decrease in Symptoms in the three years prior to STIC. However, the statistically significant interaction between Time in Period and Time indicated that Symptoms did not continue to decrease over the three years post-STIC; instead, they stabilized. The hypothesis regarding the reduction of Symptoms after the STIC implementation was thus rejected.

**Table 2:** Summaries of the Final Multilevel Model regarding Symptoms (HoNOS) and Quality of Life (MANSA)

	Symptoms	*p*	Quality of Life	*p*
	*Estimate*		*Estimate*	
Intercept	8.61	< .001	59.26	< .001
Time in period	− .0027	< .001	.0004	.448
Period	− 1.547	< .001	− .1546	.740
Time in period *period	.0024	< .001	− .0008	.291
Registration duration	− .0002	.037	.0006	< .001
Comorbidity	2.307	< .001	− 3.301	< .001
Gender	.591	.069	− 1.273	.028
Registration duration *time	1.431E-7*	.071		

^*^ E-refers to the number of decimal places the comma should be moved to the left, i.e. 1.431E-7 = 0.00000001431

Neither was our hypothesis confirmed regarding Quality of Life. There was no statistically significant main effect of Time in Period, which indicated that Quality of Life was stable during the three years prior to STIC. Also, there was no statistically significant interaction between Time and Time in Period, showing that there was no improvement of Quality of Life during the three years post-STIC.

### Representativeness of the pre-STIC short-term cure group

Of the 1918 patients included, 944 (48%) completed both pre- and post-OQ-45 measurements, and were therefore included in the analyses. Sample characteristics of the pre-STIC completers are displayed in Table 3. Compared to the non-completers, the completers differed statistically significant in the type of DSM-V classifications (*χ*2(14) = 109.589, *p* < 0.001), the number of diagnostic contacts (*U* = 362,235.500, *p* < 0.001), the number of treatment contacts (*χ*2(1) = 111.597, *p* < 0.001), the number of no-shows (*U* = 507052, *p* < 0.001), mean age (*t*(1887.985) = 2.122, *p* = 0.034), and the average treatment duration (*t*(1916) = 5.733, *p* < 0.001). Relatively more completers were diagnosed with anxiety and depressive disorder (see Table 3), and relatively less completers were diagnosed with a personality disorder than non-completers (10.2, 33.2, and 19.7% resp.). Further, completers had more diagnostic and treatment contacts, fewer no-shows, were older, and had a longer treatment duration (see Table 3) than non-completers (diagnostic *M* = 4.50, *SD* = 2.90; treatment *M* = 24.04, *SD* = 46.04; no shows *M* = 2.06, *SD* = 2.69; age *M* = 36.35, *SD* = 12.28; treatment duration *M* = 264.57, *SD* = 191.03). There were no statistically significant differences in gender, the number of crisis contacts, and the number of indirect contacts across the groups (all *p’s* > 0.05).

**Table 3:** Demographic and Clinical Characteristics of the Pre- and Post-STIC Cure Groups: Statistical Comparison with p-values

	Pre-STIC group N = 944		Post-STIC group N = 544		Test results^2^
	% (n)	*M (SD)*	% (n)	*M (SD)*	
Classification^**^					*χ*^2^(14) = 48.631, *p* < .001
Depressive disorders	39.9 (377)		40.1 (218)		
Trauma- and stressor-related disorders	12.4 (117)		12.3 (67)		
Personality disorders	13.8 (130)		8.5 (46)		
Anxiety disorders	20.3 (192)	:	17.6 (96)		
Eating disorders	5.3 (50)		5.3 (29)		
Other	8.3 (78)		16.2 (88)		
Gender (% male)	37.0 (349)		40.3 (219)		*p* = .209
Age		37.6 (13.45)		37.74 (14.08)	*p* = .850
Treatment duration^**^		313.35 (181.31)		349.86 (201.53)	*t*(1037.714) = -3.49, *p* < .001
Number of treatment contacts^*^		39.32 (65.23)		32.96 (44.69)	*χ*^2^(1) = 10.44, *p* = .001
Number of diagnostic contacts^1**^		5.50 (3.09)		3.92 (2.31)	*U* = 170,483.500, *p* < .001
Number of crisis contacts^**^		1.10 (5.55)		.35 (2.09)	*χ*^2^(1) = 155.728, *p* < .001
Number of no shows^1*^		1.55 (2.21)		1.36 (2.27)	*U* = 238,537, *p* = .016
Number of indirect contacts		7.33 (19.02)		6.77 (14.50)	p = .169

^1^For these variables non-parametric tests were used. We chose to display *M* and *SD* instead of Mean Rank or median in order to improve readability

^2^Test statistics are only reported for differences that achieve statistical significance

^*^p < .05

^**^p < .001

### Representativeness post-STIC short-term cure group

Of the 2939 patients included in the post-STIC short-term cure group, 544 (18.5%) had both pre- and post-OQ-45 measurements and were therefore included in the analyses. Sample characteristics of the post-STIC completers are displayed in Table 3. Compared to the non-completers, completers differed statistically significant in the distribution of type of DSM-V classifications (*χ2*(15) = 94.123, *p* < 0.001), the number of diagnostic contacts (*U* = 505,228, *p* < 0.001), the number of treatment contacts (*χ*2(1) = 157.917, *p* < 0.001), and average treatment duration (*t*(2927) = 10.242, *p* < 0.001). Relatively more completers were diagnosed with anxiety and depressive disorder (see Table 3) in comparison to the non-completers (13.6% and 29.8% resp.). Further, the completers had more diagnostic and treatment contacts than the non-completers (diagnostic *M* = 3.09, *SD* = 2.11; treatment *M* = 18.54, *SD* = 42.54; duration *M* = 253.87, *SD* = 196.257). There were no statistically significant differences in the number of crisis contacts, the number of no-shows, the number of indirect contacts, mean age, and gender (all *p’s* > 0.05).

### Comparability between the pre- and post-STIC cure groups

Table 3 displays demographic and clinical characteristics for the pre- (N = 944) and post-STIC cure groups (N = 544). The pre- and post-STIC groups differed significantly from each other in terms of the number of diagnostic contacts, the number of crisis contacts, the number of no-shows, and the average treatment duration. The post-STIC group had fewer treatment, diagnostic and crisis contacts, fewer no-shows, and longer treatment duration than the pre-STIC group. The distribution of DSM-V classifications also differed significantly, with a relatively higher proportion of patients diagnosed with personality and anxiety disorders in the pre-STIC group compared to the post-STIC group. Conversely, the percentage of patients with other disorders, particularly neurobiological developmental disorders, substance-related and addictive disorders, and somatic symptom disorders, was higher in the post- than in the pre-STIC group. Gender distribution and mean age did not statistically significant differ between the pre- and post-STIC groups (see Table 3).

### Results of the pre- and post-STIC cure groups

Table 4 presents pre- and post OQ-45 (subscale) mean scores, standard deviations and statistical test results for the pre- and post STIC cure groups. There was a statistically significant main effect of Time on Symptom Distress (partial *η*^*2*^ = 0.503), indicating a decrease in Symptom Distress for both groups from pre- to post-treatment. As expected, there was no statistically significant interaction-effect between Time and Group, suggesting comparable symptom reduction for both groups.

**Table 4:** Mean (M) and Standard Deviations (SD) of the Pre- and Post-STIC Cure Groups: Pre- and Post-treatment Measurements of Interpersonal Relations, Social Role and Symptomatic Distress, with Mixed ANOVA results

	Pre-STIC group n = 944				Post-STIC group n = 544				Test results	
	Pre		Post		Pre		Post		Time time*group	
	*M*	*SD*	*M*	*SD*	*M*	*SD*	*M*	*SD*		
Symptomatic distress	55.8	14.1	38.2	17.6	55.1	14.3	36.3	16.6	*F*(1,1486) = 1502.3, *p* < .001	*F*(1,1486) = 1.643, *p* = .200
Interpersonal relations	17.3	6.7	12.6	6.9	16.8	6.4	11.7	6.4	*F*(1,1486) = 788.88, *p* < .001	*F*(1,1486) = .808, *p* = .369
Social role	14.0	5.3	10.5	5.1	13.8	5.7	9.9	4.7	F(1,1486) = 532.32, p < .001	F(1,1486 = 1.24, p = .265

There was a statistically significant main effect of Time on Interpersonal Relations (partial *η*^*2*^ = 0.347), indicating an improvement in Interpersonal Relations from pre- to post-treatment, represented by decreasing scores on this subscale (see Table 4). There was no statistically significant interaction-effect between Time and Group. Contrary to the hypothesis, there was no greater improvement in Interpersonal Relations in the post-STIC group compared to the pre-STIC group. In addition, there was a statistically significant main effect of Time on Social Role (partial *η*^*2*^ = 0.289). As can be seen in Table 4, there was a reduction in scores for both groups, indicating an improvement in Social Role. However, there was no statistically significant interaction-effect between Time and Group on Social Role. Contrary to our hypothesis, there was no greater improvement in Social Role post-STIC compared to pre-STIC. 

## Discussion

In the present study we evaluated STIC by comparing patient outcomes before and after its implementation. Broadly, it can be stated that outcomes are highly similar before and after the introduction of STIC, both for patients with a relatively short and finite treatment duration as well as for patients receiving extended periods of care. More specifically, the latter group of patients, mostly diagnosed with psychotic disorder, do not seem to benefit in terms of symptom recovery in the period after STIC. Contrary, their symptoms improve slightly in the period prior to STIC, and stabilize during the period after, finding no support for our hypothesis regarding symptom recovery for the long-term care group. Neither do we find support for our hypothesis regarding quality of life, which seems to be stable during both periods before and after the implementation of STIC.

However, in accordance with our hypothesis, patients with finite treatments of maximum three years, mainly diagnosed with depressive, anxiety or personality disorder, improve equally in terms of symptoms before and after STIC. Contrary to our hypothesis, these patients do not improve more, but equally in terms of social recovery before and after STIC. These similar gains across all recovery domains for patients completing treatment after STIC (as compared to before) are, however, achieved with *fewer* treatment contacts. To comprehend these results, it is important to note that the implementation of STIC is foremost a policy direction, and therefore, in first instance, exerts influence on the context in which treatments are delivered and not *how* treatments are delivered. Changing the context may be an important condition for improving treatment quality, but detecting an effect of such an organizational change on patient level will probably require more time than the current three-year study period. Regardless, possible explanations for these ((un)expected) findings will be discussed below.

The chronicity and high degree of illness severity of the current long-term care group may partly explain the absence of symptom reduction. Stability in outcomes for these patients is a recurrent finding within our organization, but also a known phenomenon in data of other organizations collected through benchmarking efforts (Stichting Benchmark GGZ) and in previous research on the FACT population (e.g. [15]). These stable outcomes are not surprising given that until quite recently ‘stabilization of symptoms’ along with a functional adaptation of these persons to society was defined as the highest achievable treatment goal for these ‘chronic’ patients [17]. In the current study, however, it was hypothesized that for the long-term care group an increased access to specialized treatments for certain disorders, like trauma or depression, could bring some relief in terms of symptoms (and consequently quality of life). Since type of treatment was not registered during the study period, we were not able to assess whether the number of specialized treatments actually increased after the STIC implementation. And if STIC, thus, was implemented properly. Against this context, the lack of symptom reduction could be simply a result of patients not receiving more specialized treatments after the introduction of STIC as compared to before. In a follow-up study it would be advisable to monitor the type of treatments being delivered in order to increase the internal validity of the study.

It is likely that the same explanation applies to the finding that patients with a shorter and more intensive treatment program did not improve more in terms of social recovery after STIC as compared to before. As it was not possible to track the type of treatment these patients had received, it remains unclear if patients in the post-STIC period actually benefitted from an increased access to social interventions. Moreover, the outbreak of COVID-19 during the post-STIC period could also, at least in part, explain the absence of the expected findings regarding social recovery. COVID-19 negatively affected the collaboration with community partnerships which may have hindered further social participation of patients who received treatment after the implementation of STIC.

While unexpected, the slight improvement in symptoms in the long-term group prior to the STIC implementation (instead of after) does not necessarily suggest the ineffectiveness of STIC. With a maximum of three datapoints per patient within each period (pre- and post-STIC), the study may have missed higher-order symptom fluctuations. Although long-term stability in symptoms is a well-known phenomenon in this patient group, slight fluctuations are probably not uncommon.

Noteworthy is the finding that improvement in terms of symptomatic and social recovery in the post-STIC short-term cure group was achieved with fewer treatment contacts than in the equivalent pre-STIC group. Additionally, there was a reduction in the variation of treatment contacts post-STIC compared to pre-STIC. Alongside offering integrated care with evidence-based and/or practice-based treatments, another main objective of STIC is to provide targeted treatment. Through close consultation between clinician and patient, choices are being made about treatment goals and interventions, and agreements regarding the term for treating the complaints. This approach may have facilitated a more efficient treatment process, resulting in fewer treatment contacts. Moreover, if an increasing number of clinicians applied this method, leading to greater uniformity, it could explain the reduced variation in number of treatment contacts post-STIC. While the observation of fewer treatment, diagnostic and crisis contacts after the implementation of STIC could indirectly suggest potential cost savings, it is advisable for future research to include a cost-effectiveness analysis to thoroughly assess this aspect.

The questionable representativeness of the short-term cure groups in the pre- and post-STIC period may have biased the results in numerous, but unknown, ways. This moderate to poor representativeness in both periods is mostly due to the high percentage of patients without a complete OQ-45 set, the so-called non-responders. Probably most of these patients didn’t fill in the questionnaire (by the end of treatment) because of their short treatment duration, as can be also seen from their low number of treatment contacts as compared to patients with a complete OQ-45 set (see Results). This possible lack of representativeness as a result of nonresponse is difficult to overcome, but it highlights the importance of monitoring clients’ different recovery domains throughout treatment in order to evaluate if STIC meets its objectives.

A final note should be made on the notion of recovery, which has become an even more prominent topic with the implementation of STIC than it was before. As stated in the introduction, recovery is increasingly understood as a process of well-being regardless the degree of disability and distress of the individual. According to this view, patients can overcome the consequences of having a mental illness, such as social exclusion, job loss, poor housing conditions, stigma, loss of valued social roles and identity, by defining themselves apart from illness and by (re)gaining control [3, 8]. However appealing, encouraging and optimistic this view of recovery is, it often remains unclear–also in the current study-how or in the degree to which it’s principles are operationalized into actual clinical practices. Although it was beyond the scope of this study, it would be advisable in future research to operationalize the recovery principles in order to better understand their merit in relation to clients’ recovery process.

In sum, the treatment outcomes of patients within a specialized mental health institution seem to be highly comparable before and after the STIC implementation. For the short-term cure patients similar improvements are achieved in terms of symptomatic and social recovery before and after STIC, but with fewer treatment contacts post-STIC, potentially indicative for more efficiency during the treatment process. Future research should consider evaluating cost-effectiveness. For the long-term care patients, their outcomes in terms of symptomatic recovery and quality of life are predominantly stable before and after STIC. Possible explanations for the absence of the expected findings are the restricted study duration and limited insight into process outcomes, i.e. type of treatment and how recovery principles are operationalized and assessed. Gaining insight into these processes, could provide tools to better implement changes.

## Data Availability

Data is located at a secure data server at GGZ NHN. Anonymized data is available upon request by contacting onderzoek@ggz-nhn.nl.

## Footnotes

1 The decision to include patients with a minimum of 14 days between pre- and post-measurements and at least two contacts was influenced by both substantive and practical considerations. Mental health professionals provided valuable insight, noting that some patients require only 2 or 3 sessions to clarify their issues and develop simple coping strategies. While it may be debated whether these patients belong to specialized mental health care, they are nonetheless part of our patient population. From a pragmatic point of view, the time intervals between pre- and post-measurements, including those less than 30 days, are equally distributed across both the pre- and post-STIC groups, reducing the likelihood of explaining potential differences between the groups.”.

2 Indirect contacts mostly refer to multidisciplinary consultation meetings in which the progress of patients is discussed in absence of these patients.
